# (1*E*,2*E*)-1,2-Bis(2,2-diphenyl­hydrazin-1-yl­idene)ethane

**DOI:** 10.1107/S1600536810032198

**Published:** 2010-08-18

**Authors:** Angel Mendoza, Blanca M. Cabrera-Vivas, Ruth Meléndrez-Luevano, Juan C. Ramírez, Marcos Flores-Alamo

**Affiliations:** a Centro de Química, ICUAP, Benemérita Universidad Autónoma de Puebla, Puebla, Pue., Mexico; b Facultad de Ciencias Químicas, Benemérita Universidad Autónoma de Puebla, Puebla, Pue., Mexico; c Facultad de Química, Universidad Nacional Autónoma de México, 04510 México DF, Mexico

## Abstract

In the crystal structure of the title compound, C_26_H_22_N_4_, the mol­ecule is located on an inversion centre and shows an *E* configuration with respect to each C=N bond. The dihedral angle between the phenyl rings in the diphenyl­hydrazone group is 83.69 (11)°. These two rings make dihedral angles of 30.53 (15) and 84.53 (16)° with the central N—N=C—C=N—N dihydrazonoethane plane. Inter­molecular C—H⋯π inter­actions are observed.

## Related literature

For applications of hydrazones, see: Angell *et al.* (2006 ▶); Ibañez *et al.* (2002 ▶). For related structures, see: Clulow *et al.* (2008 ▶); Mendoza *et al.* (2010 ▶). For bond-length data, see: Allen *et al.* (1987 ▶).
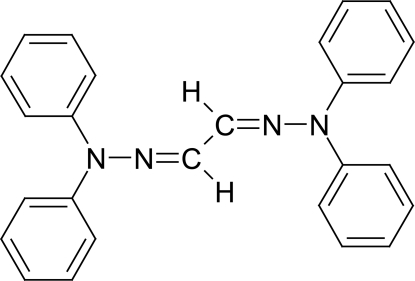


## Experimental

### 

#### Crystal data

C_26_H_22_N_4_
*M* *_r_* = 390.48
Monoclinic, 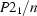

*a* = 12.2210 (19) Å
*b* = 5.612 (1) Å
*c* = 15.731 (3) Å
β = 103.924 (16)°
*V* = 1047.2 (3) Å^3^
*Z* = 2
Cu *K*α radiation
μ = 0.58 mm^−1^
*T* = 298 K
0.19 × 0.11 × 0.05 mm

#### Data collection

Oxford Xcalibur Atlas Gemini diffractometer
Absorption correction: analytical (*CrysAlis PRO*; Oxford Diffraction, 2010 ▶) *T*_min_ = 0.978, *T*_max_ = 0.993
3621 measured reflections
1892 independent reflections
1163 reflections with *I* > 2σ(*I*)
*R*_int_ = 0.038

#### Refinement

*R*[*F* ^2^ > 2σ(*F* ^2^)] = 0.044
*wR*(*F* ^2^) = 0.113
*S* = 1.01
1892 reflections
137 parameters
H-atom parameters constrained
Δρ_max_ = 0.13 e Å^−3^
Δρ_min_ = −0.14 e Å^−3^

### 

Data collection: *CrysAlis CCD* (Oxford Diffraction, 2009 ▶); cell refinement: *CrysAlis RED* (Oxford Diffraction, 2009 ▶); data reduction: *CrysAlis RED*; program(s) used to solve structure: *SHELXS97* (Sheldrick, 2008 ▶); program(s) used to refine structure: *SHELXL97* (Sheldrick, 2008 ▶); molecular graphics: *ORTEP-3 for Windows* (Farrugia, 1997 ▶); software used to prepare material for publication: *WinGX* (Farrugia, 1999 ▶).

## Supplementary Material

Crystal structure: contains datablocks I, global. DOI: 10.1107/S1600536810032198/is2589sup1.cif

Structure factors: contains datablocks I. DOI: 10.1107/S1600536810032198/is2589Isup2.hkl

Additional supplementary materials:  crystallographic information; 3D view; checkCIF report

## Figures and Tables

**Table 1:** Hydrogen-bond geometry (Å, °) *Cg*1 and *Cg*2 are the centroids of the C1–C6 and C7–C12 rings, respectively.

*D*—H⋯*A*	*D*—H	H⋯*A*	*D*⋯*A*	*D*—H⋯*A*
C3—H3⋯*Cg*2^i^	0.93	2.85	3.728 (3)	159
C8—H8⋯*Cg*1^ii^	0.93	2.88	3.785 (3)	164

Symmetry codes: (i) 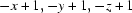; (ii) 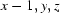.

**Fig. 1.**
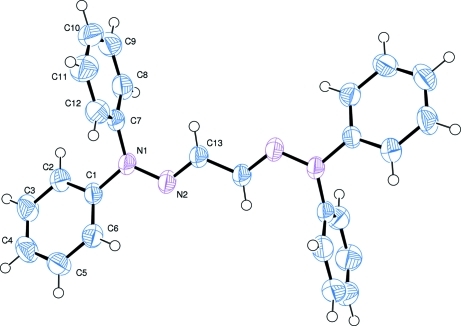
The molecular structure of compound I, with atom labels and 50% probability displacement ellipsoids for non-H atoms.

## supplementary crystallographic information

### Comment

Among the most interesting applications of hydrazones, molecular sensing is worth mentioning. They are being used widely to detect chemical and biological species (Angell *et al.*, 2006). Also, hydrazones are being applied as plasticizer agents, polymerization initiators and antioxidants (Ibañez *et al.*, 2002). There are pigments, as 1-fenilazo-2-naftol, that show an azo/hydrazone tautomery in which the main tautomer exist as hydrazone form.

The asymmetric unit of the title compound I consist of C_13_H_11_N_2_ with a *Z'* = 0.5 showing a centrosymmetrical structure. The compound I (C_26_H_22_N_4_) present an *E* configuration for each C═N double bond (Fig. 1), with *N*,*N*-diphenyl group opposite to second C═N group. The molecule shows a non-planar structure for phenyl rings respect to N—N group, with a torsion angle between them C2—C1—N1—C7 = 46.6 (3)°. The torsion angle of phenyl ring C1/C2/C3/C4/C5/C6 to N—N═C group is -173.48 (18)°, and the other ring C7/C8/C9/C10/C11/C12 shows a torsion angle of -14.9 (3)° to the same group. The N—N distance [1.364 (2) Å] is shorter than found in free diphenylhydrazine [1.418 (2) Å] (Clulow *et al.*, 2008). Imine bond distance, N2═C13 [1.287 (2) Å], is longer than N═C typical bond (Allen *et al.*, 1987), but similar [1.286 (3) Å] to related structures with *N*,*N*-diphenylhidrazone group (Mendoza *et al.*, 2010).

### Experimental

*N*,*N*-diphenylhydrazine (2.74 mg, 12.4 mmol) was dissolved in ethanol and acetic acid (0.5 ml) was added slowly into this solution while stirring. Glyoxal (300 mg, 5.1 mmol) was added drop by drop into the above solution with strong stirring and the resulting mixture was kept at atmospheric temperature until it became yellow solution. After three hours, the amber solution turns to be precipitated. The mixture was separated with filtration in vacuum system and the precipitate was washed three times with cold methanol. Recrystallization was performed several times with acetonitrile, to obtain needle crystals suitable for X-ray analysis. Yield: 1.79 g (90%) at 25 °C, mp. 185–189 °C. FT–IR (film): (cm^-1^): 3062 ν(C—H), 1750–2000 ν(Ph), 1591, 1544, 1490 ν(C═N). EI–MS: m/*z* 390 *M*^+^.

### Refinement

H atoms were placed in geometrical idealized positions (C—H = 0.93 Å) and refined as riding on their parent atoms, with *U*_iso_(H) = 1.2*U*_eq_(C).

### Crystal data

**Table:**

C_26_H_22_N_4_	*F*(000) = 412
*M**_r_* = 390.48	*D*_x_ = 1.238 Mg m^−^^3^
Monoclinic, *P*2_1_/*n*	Cu *K*α radiation, λ = 1.5418 Å
*a* = 12.2210 (19) Å	Cell parameters from 864 reflections
*b* = 5.612 (1) Å	θ = 3.7–68.0°
*c* = 15.731 (3) Å	µ = 0.58 mm^−^^1^
β = 103.924 (16)°	*T* = 298 K
*V* = 1047.2 (3) Å^3^	Prism, colourless
*Z* = 2	0.19 × 0.11 × 0.05 mm

### Data collection

**Table:**

Oxford Xcalibur Atlas Gemini diffractometer	1892 independent reflections
graphite	1163 reflections with *I* > 2σ(*I*)
Detector resolution: 10.4685 pixels mm^-1^	*R*_int_ = 0.038
ω scans	θ_max_ = 68.2°, θ_min_ = 4.1°
Absorption correction: analytical (*CrysAlis PRO*; Oxford Diffraction, 2010)	*h* = −14→14
*T*_min_ = 0.978, *T*_max_ = 0.993	*k* = −4→6
3621 measured reflections	*l* = −18→10

### Refinement

**Table:**

Refinement on *F*^2^	Secondary atom site location: difference Fourier map
Least-squares matrix: full	Hydrogen site location: inferred from neighbouring sites
*R*[*F*^2^ > 2σ(*F*^2^)] = 0.044	H-atom parameters constrained
*wR*(*F*^2^) = 0.113	*w* = 1/[σ^2^(*F*_o_^2^) + (0.0496*P*)^2^ + 0.0674*P*] where *P* = (*F*_o_^2^ + 2*F*_c_^2^)/3
*S* = 1.01	(Δ/σ)_max_ < 0.001
1892 reflections	Δρ_max_ = 0.13 e Å^−^^3^
137 parameters	Δρ_min_ = −0.14 e Å^−^^3^
0 restraints	Extinction correction: *SHELXL97* (Sheldrick, 2008), Fc^*^=kFc[1+0.001xFc^2^λ^3^/sin(2θ)]^-1/4^
Primary atom site location: structure-invariant direct methods	Extinction coefficient: 0.0134 (9)

### Special details

**Table:**

Geometry. All s.u.'s (except the s.u. in the dihedral angle between two l.s. planes) are estimated using the full covariance matrix. The cell s.u.'s are taken into account individually in the estimation of s.u.'s in distances, angles and torsion angles; correlations between s.u.'s in cell parameters are only used when they are defined by crystal symmetry. An approximate (isotropic) treatment of cell s.u.'s is used for estimating s.u.'s involving l.s. planes.
Refinement. Refinement of *F*^2^ against ALL reflections. The weighted *R*-factor *wR* and goodness of fit *S* are based on *F*^2^, conventional *R*-factors *R* are based on *F*, with *F* set to zero for negative *F*^2^. The threshold expression of *F*^2^ > 2σ(*F*^2^) is used only for calculating *R*-factors(gt) *etc*. and is not relevant to the choice of reflections for refinement. *R*-factors based on *F*^2^ are statistically about twice as large as those based on *F*, and *R*- factors based on ALL data will be even larger.

### Fractional atomic coordinates and isotropic or equivalent isotropic displacement parameters (Å^2^)

**Table:**

	*x*	*y*	*z*	*U*_iso_*/*U*_eq_	
N2	0.09341 (13)	0.1892 (3)	0.46419 (10)	0.0451 (5)	
N1	0.20416 (13)	0.2544 (3)	0.48657 (10)	0.0481 (5)	
C1	0.23555 (16)	0.4384 (4)	0.43534 (12)	0.0421 (5)	
C13	0.05715 (15)	0.0396 (4)	0.51307 (13)	0.0451 (5)	
H13	0.1046	−0.0161	0.5646	0.054*	
C12	0.27665 (18)	0.3696 (4)	0.63743 (14)	0.0541 (6)	
H12	0.2307	0.5037	0.6265	0.065*	
C7	0.27471 (15)	0.2080 (4)	0.57158 (12)	0.0415 (5)	
C6	0.16000 (18)	0.6077 (4)	0.39339 (13)	0.0494 (5)	
H6	0.0859	0.6044	0.3988	0.059*	
C2	0.34652 (17)	0.4494 (4)	0.42837 (13)	0.0532 (6)	
H2	0.3988	0.3382	0.4574	0.064*	
C5	0.1938 (2)	0.7827 (4)	0.34321 (14)	0.0587 (6)	
H5	0.1422	0.8956	0.3147	0.07*	
C8	0.33966 (18)	0.0069 (4)	0.58755 (15)	0.0572 (6)	
H8	0.3374	−0.1049	0.5435	0.069*	
C3	0.3793 (2)	0.6255 (4)	0.37837 (15)	0.0623 (7)	
H3	0.4538	0.6319	0.3739	0.075*	
C4	0.3036 (2)	0.7904 (4)	0.33537 (15)	0.0640 (7)	
H4	0.3261	0.9069	0.3011	0.077*	
C10	0.4116 (2)	0.1382 (6)	0.73586 (17)	0.0726 (8)	
H10	0.4585	0.115	0.7913	0.087*	
C9	0.40936 (19)	−0.0277 (5)	0.67093 (19)	0.0722 (8)	
H9	0.4544	−0.163	0.6827	0.087*	
C11	0.3458 (2)	0.3350 (5)	0.71930 (15)	0.0708 (8)	
H11	0.3473	0.4465	0.7633	0.085*	

### Atomic displacement parameters (Å^2^)

**Table:**

	*U*^11^	*U*^22^	*U*^33^	*U*^12^	*U*^13^	*U*^23^
N2	0.0382 (9)	0.0534 (11)	0.0436 (10)	−0.0137 (8)	0.0099 (7)	−0.0026 (8)
N1	0.0372 (9)	0.0609 (11)	0.0446 (10)	−0.0163 (8)	0.0066 (7)	0.0080 (9)
C1	0.0439 (11)	0.0468 (12)	0.0357 (10)	−0.0134 (10)	0.0096 (8)	−0.0006 (9)
C13	0.0391 (10)	0.0537 (13)	0.0428 (11)	−0.0106 (10)	0.0101 (9)	0.0036 (11)
C12	0.0544 (13)	0.0597 (14)	0.0500 (13)	−0.0014 (12)	0.0161 (10)	0.0011 (12)
C7	0.0352 (10)	0.0470 (12)	0.0438 (11)	−0.0101 (10)	0.0125 (8)	0.0036 (10)
C6	0.0480 (12)	0.0532 (13)	0.0470 (12)	−0.0083 (11)	0.0113 (10)	−0.0035 (11)
C2	0.0461 (12)	0.0587 (14)	0.0564 (13)	−0.0081 (11)	0.0157 (10)	0.0069 (11)
C5	0.0705 (16)	0.0501 (14)	0.0523 (13)	−0.0041 (12)	0.0087 (11)	0.0069 (11)
C8	0.0512 (13)	0.0509 (13)	0.0713 (16)	−0.0074 (12)	0.0184 (12)	0.0017 (13)
C3	0.0574 (14)	0.0731 (16)	0.0640 (15)	−0.0158 (13)	0.0297 (12)	0.0068 (13)
C4	0.0806 (17)	0.0612 (16)	0.0540 (14)	−0.0189 (14)	0.0234 (12)	0.0076 (12)
C10	0.0544 (14)	0.102 (2)	0.0554 (16)	−0.0156 (16)	0.0024 (12)	0.0226 (16)
C9	0.0484 (13)	0.0676 (17)	0.098 (2)	0.0028 (13)	0.0116 (14)	0.0304 (16)
C11	0.0731 (16)	0.092 (2)	0.0463 (14)	−0.0098 (16)	0.0125 (12)	−0.0014 (14)

### Geometric parameters (Å, °)

**Table:**

N2—C13	1.287 (2)	C2—C3	1.381 (3)
N2—N1	1.364 (2)	C2—H2	0.93
N1—C1	1.418 (2)	C5—C4	1.377 (3)
N1—C7	1.430 (2)	C5—H5	0.93
C1—C6	1.377 (3)	C8—C9	1.395 (3)
C1—C2	1.388 (3)	C8—H8	0.93
C13—C13^i^	1.429 (4)	C3—C4	1.367 (3)
C13—H13	0.93	C3—H3	0.93
C12—C7	1.373 (3)	C4—H4	0.93
C12—C11	1.373 (3)	C10—C11	1.354 (3)
C12—H12	0.93	C10—C9	1.377 (4)
C7—C8	1.368 (3)	C10—H10	0.93
C6—C5	1.384 (3)	C9—H9	0.93
C6—H6	0.93	C11—H11	0.93
			
C13—N2—N1	118.93 (16)	C4—C5—C6	120.3 (2)
N2—N1—C1	115.85 (16)	C4—C5—H5	119.9
N2—N1—C7	122.01 (14)	C6—C5—H5	119.9
C1—N1—C7	118.63 (15)	C7—C8—C9	118.9 (2)
C6—C1—C2	119.11 (19)	C7—C8—H8	120.5
C6—C1—N1	122.18 (18)	C9—C8—H8	120.5
C2—C1—N1	118.71 (19)	C4—C3—C2	120.8 (2)
N2—C13—C13^i^	119.0 (2)	C4—C3—H3	119.6
N2—C13—H13	120.5	C2—C3—H3	119.6
C13^i^—C13—H13	120.5	C3—C4—C5	119.5 (2)
C7—C12—C11	120.6 (2)	C3—C4—H4	120.2
C7—C12—H12	119.7	C5—C4—H4	120.2
C11—C12—H12	119.7	C11—C10—C9	120.2 (2)
C8—C7—C12	120.2 (2)	C11—C10—H10	119.9
C8—C7—N1	120.98 (19)	C9—C10—H10	119.9
C12—C7—N1	118.86 (19)	C10—C9—C8	120.1 (2)
C1—C6—C5	120.3 (2)	C10—C9—H9	119.9
C1—C6—H6	119.8	C8—C9—H9	119.9
C5—C6—H6	119.8	C10—C11—C12	119.9 (2)
C3—C2—C1	120.0 (2)	C10—C11—H11	120.1
C3—C2—H2	120	C12—C11—H11	120.1
C1—C2—H2	120		
			
C13—N2—N1—C1	−173.48 (18)	N1—C1—C6—C5	−179.26 (18)
C13—N2—N1—C7	−14.9 (3)	C6—C1—C2—C3	−1.3 (3)
N2—N1—C1—C6	26.8 (3)	N1—C1—C2—C3	179.48 (18)
C7—N1—C1—C6	−132.5 (2)	C1—C6—C5—C4	−0.6 (3)
N2—N1—C1—C2	−154.06 (18)	C12—C7—C8—C9	−1.5 (3)
C7—N1—C1—C2	46.6 (3)	N1—C7—C8—C9	178.36 (18)
N1—N2—C13—C13^i^	−176.3 (2)	C1—C2—C3—C4	0.0 (3)
C11—C12—C7—C8	1.7 (3)	C2—C3—C4—C5	1.0 (4)
C11—C12—C7—N1	−178.09 (18)	C6—C5—C4—C3	−0.8 (3)
N2—N1—C7—C8	93.9 (2)	C11—C10—C9—C8	0.4 (4)
C1—N1—C7—C8	−108.1 (2)	C7—C8—C9—C10	0.4 (3)
N2—N1—C7—C12	−86.3 (2)	C9—C10—C11—C12	−0.1 (4)
C1—N1—C7—C12	71.7 (2)	C7—C12—C11—C10	−0.9 (3)
C2—C1—C6—C5	1.6 (3)		

Symmetry codes: (i) −*x*, −*y*, −*z*+1.

### Hydrogen-bond geometry (Å, °)

**Table:**

*D*—H···*A*	*D*—H	H···*A*	*D*···*A*	*D*—H···*A*
C3—H3···Cg2^ii^	0.93	2.85	3.728 (3)	159
C8—H8···Cg1^iii^	0.93	2.88	3.785 (3)	164

Symmetry codes: (ii) −*x*+1, −*y*+1, −*z*+1; (iii) *x*−1, *y*, *z*.
